# ADP-Based Fault-Tolerant Control with Stability Guarantee for Nonlinear Systems

**DOI:** 10.3390/e27101028

**Published:** 2025-10-01

**Authors:** Luojia Liu, Junhong Lv, Haowei Lin, Ruidian Zhan, Liming Wu

**Affiliations:** School of Advanced Manufacturing, Guangdong University of Technology, Jieyang 515200, China; 3222008670@mail2.gdut.edu.cn (L.L.); 3122009014@mail2.gdut.edu.cn (J.L.); rd.zhan@gdut.edu.cn (R.Z.); jkyjs@gdut.edu.cn (L.W.)

**Keywords:** adaptive dynamic programming, actuator fault, fault observer, Lyapunov stability

## Abstract

This paper develops the stability-guaranteed adaptive dynamic programming (ADP)-based fault tolerant control (FTC) for nonlinear systems with an actuator fault. Firstly, a fault observer is designed to identify the unknown actuator fault. Then, a critic neural network (NN) is built to approximate the optimal control of the nominal system. Meanwhile, a stability-aware weight update mechanism is proposed based on the Lyapunov stability theorem to relax the restriction of the initial admissible control on the system stability. By integrating the nominal optimal control and the fault estimation, the stability-guaranteed ADP-based FTC is developed to eliminate the influence of the actuator fault. Furthermore, the observer errors, critic NN weight estimation errors, and the closed-loop system are all shown to exhibit uniform ultimate boundedness by using Lyapunov’s direct method. Finally, simulation examples are given to demonstrate the validity of the proposed method.

## 1. Introduction

The increasing scale and complexity of modern industrial systems pose significant challenges to optimal controller design. Adaptive dynamic programming (ADP) has emerged as an effective tool of optimal control, particularly for high-dimensional nonlinear systems where exact mathematical models are unavailable. ADP derives the optimal control by integrating the technique of reinforcement learning (RL), dynamic programming, and neural networks (NNs). With years of developments, ADP has found widespread application in complex nonlinear systems, including robust control [1], fault tolerant control (FTC) [2], zero-sum games [3], actuator saturation [4], and so on.

Value iteration (VI) and policy iteration (PI) are two basic algorithms of ADP. Based on VI, the value function converges to the optima monotonically without requiring an initial admissible policy (IAP). The convergence analysis of continuous VI was presented in [5]. It was proved that the value function initialized with an arbitrary positive semidefinite function can converge to a neighborhood of the optimum. Ha et al. [6] improved the VI performance by developing a novel adaptive critic design with a discount factor, which accelerates the learning formulation and relaxes conditions on the system dynamics. However, VI cannot guarantee system stability during the learning process. In contrast, PI starts with the IAP and alternates between policy evaluation and improvement while ensuring stability at each iteration. Thus, PI is generally considered more suitable for practical applications. Nonetheless, one notable limitation of PI is its reliance on an IAP, which is difficult to obtain. To address this problem, Duan et al. [7] proposed a PI-based actor–critic approach with stability guarantees for nonlinear systems, where a warm-up phase was introduced to guide the initial policy toward admissibility. Combining the advantages of VI and PI, Luo et al. [8] developed a novel ADP method by adding a parameter to balance VI and PI, which not only accelerates VI but also eliminates the need of an IAP. In [9], Guo et al. studied the event-triggered tracking control utilizing ADP with a single critic NN, where the dependence on the IAP is removed by refining the weight update mechanism.

Due to the increasing complexity of industrial systems, faults in control components are a frequent occurrence, with actuator faults being one of the most common types. Such faults can lead to a significant degradation of system reliability, safety, and stability. In response to the severe challenges posed by actuator faults, FTC has emerged as a critical technology for ensuring system safety and enhancing reliability. A variety of classical FTC strategies have been established, such as observer-based methods [10,11,12], sliding mode control (ISMC) [13,14,15], and fuzzy logic control (FLC) [16,17,18]. Recently, increasing attention has been given to ADP-based FTC methods. A number of studies have achieved success by integrating ADP with observer-based techniques. Liu [19] employs an NN-based fault observer for online fault estimation and compensation. A neuro-dynamic programming-based FTC scheme is proposed in [20], which combines a state observer with a fault observer to simultaneously estimate the state and multiple faults. This observer-based paradigm has also been extended to handle more demanding dynamics, such as state time delays [21,22] and complex multi-agent interactions [23,24]. However, their reliance on model knowledge for observer design limits their application to systems with unknown dynamics. To address this limitation, model-free approaches have become a major research focus. Lin et al. [25] proposed a data-based FTC framework which uses the particle swarm optimization algorithm to optimize the NN identifier and the critic NN for unknown nonlinear systems. More recently, fully RL methods have been developed to learn the control policy directly from system data. For instance, Zhang et al. [26] utilizes a model-free, off-policy RL algorithm to learn and deploy a differential-game-based $H_{\infty }$ fault-tolerant controller. This controller enables effective compensation for actuator faults while mitigating external disturbances. Ref. [27] integrates the reference trajectory and system state. The authors’ method learns the optimal control policy using both offline pre-training and an online actor–critic framework, which enables optimal fault-tolerant tracking control for nonlinear systems. A notable challenge in these online learning schemes is the stringent persistent excitation (PE) condition. Wu et al. [28] developed a distributed tracking control scheme based on event-triggered ADP. This scheme introduces an event-triggered mechanism to save computation and communication resources while relaxing the PE condition. These model-free strategies have also been adapted to manage complex scenarios involving multi-fault handling and input constraints [29].

It is worth mentioning that many existing ADP-based FTC methods are developed based on the PI algorithm, which completely relies on an IAP policy. However, as is mentioned above, it is extremely difficult to obtain an IAP theoretically, which limits the application of the ADP-based FTC algorithms. To address this challenge, this paper proposes a stability-guaranteed ADP-based FTC strategy that eliminates the need for the admissible policy. The key contributions of this work are described as follows:

(1) A stability-aware weight update mechanism with an auxiliary stabilizing term is proposed to evaluate real-time system stability and correct the learning process of the critic NN, which ensures the system stability in the absence of the initial stabilizing control.
(2) By designing a fault observer, the unknown actuator fault is estimated accurately and compensated based on the nominal optimal control, thereby eliminating the influence of the actuator fault.

## 2. Problem Statement and Preliminaries

### 2.1. Problem Formulation

Consider the following continuous-time nonlinear system with the actuator fault:

(1) $$\dot{x}\left(t\right)=f\left(x\left(t\right)\right)+g\left(x\left(t\right)\right)\left(u\left(x\left(t\right)\right)-f_{a}\left(t\right)\right),$$

where $x\in R^{n}$ is the system state vector, $u\in R^{m}$ is the control input vector, and $f_{a}\left(t\right)\in R^{m}$ denotes the unknown actuator fault. The function $f\left(x\right)\in R^{n}$ is a nonlinear function satisfying f(0)=0. $g\left(x\right)\in R^{n\times m}$ is the input gain function. The initial state is given by $x\left(0\right)=x_{0}$.

**Assumption** **1.** *The actuator fault $f_{a}\left(t\right)$ is unknown but bounded by a positive constant $\delta_{1}$, i.e., $∥f_{a}\left(t\right)∥\leq \delta_{1}$ [30].*

The objective of this paper is to design the FTC policy u(x(t)) for the system (1) subject to the unknown but bounded actuator fault $f_{a}\left(t\right)$ satisfying Assumption 1. While actuator faults share the input channel g(x) with matched disturbances, they are fundamentally distinct from typical external disturbances. First, actuator faults represent an internal malfunction that directly compromises the intended execution and effectiveness of the control signal, rather than being merely an additive exogenous input. This distinction necessitates the explicit fault identification and compensatory control strategies to maintain stability and recover performance. Second, actuator faults originate from random internal component failures within the system. This makes their occurrence difficult to predict and poses a far greater threat to system stability and safety than conventional disturbances.

### 2.2. Nominal Optimal Control

We first consider the nominal optimal control problem for the system in the absence of the fault, i.e., where $f_{a}\left(t\right)=0$. The nominal system dynamics become

(2) $$\;\dot{x}\left(t\right)=f\left(x\left(t\right)\right)+g\left(x\left(t\right)\right)u\left(x\left(t\right)\right).$$

The performance index for the nominal system (2) is defined as

(3) $$\;J\left(x_{0}\right)=\int_{0}^{\infty }U\left(x\left(\tau \right),u\left(\tau \right)\right)\;d\tau ,$$

where the utility function is defined by $U\left(x,u\right)=x^{⊤}Qx+u^{⊤}Ru$, with $Q\in R^{n\times n}$ and $R\in R^{m\times m}$ being constant symmetric positive-definite matrices.

**Definition** **1.** *For the nominal system (2) (i.e., system (1) with $f_{a}\left(t\right)=0$), a control policy u(x) is continuous on a compact set $\Omega \subset R^{n}$, satisfies u(0)=0, stabilizes the system (2), and results in a finite performance index $J(x_{0})$ as defined in (3) for all $x_{0}\in \Omega$, then the control policy u(x) is said to be admissible.*

Let Ψ(Ω) denote the set of admissible policies on Ω. If J(x) is continuously differentiable, the nonlinear Lyapunov equation is

(4) $$0=U\left(x,u\left(x\right)\right)+\nabla V{\left(x\right)}^{⊤}\left(f(x)+g(x)u(x)\right),$$

where V(0)=0, and $\nabla V\left(x\right)=\frac{\partial V(x)}{\partial x}$ denotes the gradient of V(x) with respect to *x*.

The Hamiltonian function is given by

(5) $$H\left(x,u,\nabla V\left(x\right)\right)=U\left(x,u\right)+\nabla V{\left(x\right)}^{⊤}\left(f(x)+g(x)u\right).$$

Using this definition, the Lyapunov Equation (4) can be simply expressed as

(6) H(x,u(x),∇V(x))=0.

The optimal control problem aims to find the control policy $u^{*}\left(x\right)\in \Psi \left(\Omega \right)$ that minimizes the performance index (3). The optimal performance index is defined as

(7) $$\;J^{*}\left(x\right)=\min_{u\in \Psi (\Omega )}\int_{0}^{\infty }U\left(x\left(\tau \right),u\left(x\left(\tau \right)\right)\right)\;d\tau ,$$

and the corresponding HJB equation is

(8) $$\;0=\min_{u\in \Psi (\Omega )}\left\{U\left(x,u\left(x\right)\right)+\nabla J^{*}{\left(x\right)}^{⊤}\left(f(x)+g(x)u(x)\right)\right\},$$

where $\nabla J^{*}\left(x\right)=\partial J^{*}\left(x\right)/\partial x$. The HJB equation can also be written using the Hamiltonian as $\min_{u\in \Psi (\Omega )}H\left(x,u\left(x\right),\nabla J^{*}\left(x\right)\right)=0$, where $\nabla J^{*}\left(x\right)$ now takes the role of ∇V(x) in the Hamiltonian definition (5). By solving the HJB equation, an optimal control policy is obtained:

(9) $$u^{*}\left(x\right)=-\frac{1}{2}R^{-1}g^{⊤}\left(x\right)\nabla J^{*}\left(x\right).$$

The optimal control $u^{*}\left(x\right)$ given by (9) and its corresponding optimal value function $J^{*}\left(x\right)$ are typically found using iterative methods. PI is a widely used ADP approach that effectively solves the HJB equation. PI conventionally alternates between two main steps: policy evaluation, in which the value function for the current control policy is determined by solving the Bellman Equation (4), and policy improvement, where the control policy is updated using the gradient of this value function to better approximate $u^{*}\left(x\right)$. However, a significant challenge in conventional PI is its requirement for an initial admissible control policy to begin the iteration. This limitation motivates the development of techniques that can operate without a predefined initial admissible control.

From the optimal control law expression (9), we can derive a useful relationship:

(10) $${\left(\nabla J^{*}\left(x\right)\right)}^{⊤}g\left(x\right)=-2{\left(u^{*}\left(x\right)\right)}^{⊤}R.$$

## 3. Stability-Guaranteed ADP-Based FTC Without IAP

### 3.1. Fault Observer Design

For the faulty system described in (1), we design the fault observer as

(11) x^˙=f^(x)+g^(x)u−f^a+L1(x−x^),

where x^∈Rn denotes the estimated system state, $L_{1}\in R^{n\times n}$ is a positive-definite observer gain matrix, and f^a∈Rm is the estimated actuator fault. The fault estimate f^a is updated using the following adaptive law:

(12) f^˙a=−L2g^⊤(x)eo,

where $L_{2}\in R^{m\times m}$ is a positive-definite gain matrix, and eo=x−x^ denotes the observation error. According to the system dynamics (2) and the observer (11), the error dynamics are derived as

(13) e˙o=f(x)+g(x)u−fa−f^(x)−g^(x)u−f^a−L1eo=f˜+g˜u−g(x)fa+g^(x)f^a−L1eo,

where f˜=fx−f^x and g˜=gx−g^x represent the observation errors of the nonlinear components f(x) and g(x), respectively.

Define $\varsigma =\overset{˜}{f}+\overset{˜}{g}\left(u-f_{a}\right)$; the error dynamics in (13) is expressed as

e˙o=ς−g^(x)(fa−f^a)−L1eo.

**Assumption** **2.** *ς is norm-bounded, i.e., $∥\varsigma ∥\leq \varsigma_{M}$, where $\varsigma_{M}$ is a positive constant [30].*

**Theorem** **1.** *For the faulty system *(1)* with Assumptions 1 and 2, the fault observation error is uniformly ultimately bounded (UUB) using the fault observer *(11)* and the adaptive update law *(12)*.*

**Proof.**  Select the Lyapunov function candidate as

(14) $$\;\Sigma_{1}=\frac{1}{2}e_{o}^{⊤}e_{o}+\frac{1}{2}{\overset{˜}{f}}_{a}^{⊤}L_{2}^{-1}{\overset{˜}{f}}_{a},$$

where f˜a=fa−f^a is the estimation error of the actuator fault. Taking the time derivative of (14) and substituting (13) yields

Σ˙1=eo⊤e˙o−f^˙a⊤L2−1f˜a=eo⊤f˜+g˜(u−fa)−g^(x)(fa−f^a)−L1eo−f^˙a⊤L2−1f˜a≤ςM∥eo∥−eo⊤g^(x)f˜a−λmin(L1)∥eo∥2−f^˙a⊤L2−1f˜a=−λmin(L1)∥eo∥−ςM∥eo∥−eo⊤g^(x)+f^˙a⊤L2−1f˜a

Substituting the adaptive law (12) into the above yields

(15) $${\dot{\Sigma }}_{1}=-\left(\lambda_{\min}\left(L_{1}\right)∥e_{o}∥-\varsigma_{M}\right)∥e_{o}∥.$$

This implies that we have ${\dot{\Sigma }}_{1}<0$ as long as $e_{o}$ lies outside the compact set $∥e_{o}∥\leq \frac{\varsigma_{M}}{\lambda_{\min}\left(L_{1}\right)}$. According to the Lyapunov stability theorem, the fault observation error is UUB. The proof is thus complete. □

**Remark** **1.** *The observer gains $L_{1}$ and $L_{2}$, as well as the disturbance bound $\varsigma_{M}$, directly affect the convergence rate and ultimate bound of the estimation error. A larger $L_{1}$ improves convergence speed, while $L_{2}$ regulates the responsiveness of fault estimation. Appropriate tuning is essential to balance estimation accuracy and robustness.*

### 3.2. Nominal Optimal Control via Critic Neural Network

NNs are known to be universal approximators for nonlinear functions. In this work, the critic NN is utilized to approximate the optimal value function $J^{*}\left(x\right)$ (7), which is the solution to the HJB Equation (8) and is typically unknown, nonlinear, and non-analytic. The optimal value function $J^{*}\left(x\right)$ can be represented by an NN as

(16) $$\;J^{*}\left(x\right)=W_{c}^{⊤}\sigma \left(x\right)+\varepsilon_{c}\left(x\right),$$

where $W_{c}\in R^{l}$ is the ideal weight vector, $\sigma \left(x\right)\in R^{l}$ is the vector of activation functions, *l* is the number of neurons in the hidden layer, and $\varepsilon_{c}\left(x\right)$ is the NN approximation error. The gradient of the optimal value function is then

(17) $$\nabla J^{*}\left(x\right)={\left(\nabla \sigma \left(x\right)\right)}^{⊤}W_{c}+\nabla \varepsilon_{c}\left(x\right),$$

where $\nabla \sigma \left(x\right)=\partial \sigma \left(x\right)/\partial x\in R^{l\times n}$. When substituting the NN approximation (17) into (8), the resulting expression can be separated into terms involving ${\left(\nabla \sigma \left(x\right)\right)}^{⊤}W_{c}$ and terms involving the approximation error gradient $\nabla \varepsilon_{c}\left(x\right)$. We define $e_{cH}$ as the sum of all terms that include $\varepsilon_{c}\left(x\right)$ or its gradient $\nabla \varepsilon_{c}\left(x\right)$, such that the HJB equation is satisfied by setting the sum of the principal part (formed with ${\left(\nabla \sigma \left(x\right)\right)}^{⊤}W_{c}$) and $e_{cH}$ to zero. Specifically, $e_{cH}$ is derived as

(18) $$\begin{array}{cc} e_{cH} & ={\left(\nabla \varepsilon_{c}\left(x\right)\right)}^{⊤}f\left(x\right)-\frac{1}{2}{\left(\nabla \varepsilon_{c}\left(x\right)\right)}^{⊤}G\left(x\right){\left(\nabla \sigma \left(x\right)\right)}^{⊤}W_{c}\\ \; & \;-\frac{1}{4}{\left(\nabla \varepsilon_{c}\left(x\right)\right)}^{⊤}G\left(x\right)\nabla \varepsilon_{c}\left(x\right), \end{array}$$

where $G\left(x\right)=g\left(x\right)R^{-1}g^{⊤}\left(x\right)$.

Since the ideal weight vector $W_{c}$ is unknown, (16) is approximated as

(19) J^*(x)=W^c⊤σ(x),

with gradient

(20) ∇J^*(x)=(∇σ(x))⊤W^c,

where W^c is the estimate of $W_{c}$.

By substituting ∇J^*(x), the approximate Hamiltonian error is defined as

(21) ec=x⊤Qx+((∇σ(x))⊤W^c)⊤f(x)−14((∇σ(x))⊤W^c)⊤G(x)((∇σ(x))⊤W^c).

Let W˜c=Wc−W^c be the weight estimation error. The Hamiltonian error $e_{c}$ can be expressed in terms of ${\overset{˜}{W}}_{c}$, $W_{c}$, and $e_{cH}$ as

(22) $$\begin{array}{cc} e_{c} & =-{\overset{˜}{W}}_{c}^{⊤}\left(\nabla \sigma \left(x\right)f\left(x\right)-\frac{1}{2}\nabla \sigma \left(x\right)G\left(x\right){\left(\nabla \sigma \left(x\right)\right)}^{⊤}W_{c}\right)\\ \; & \;-\frac{1}{4}{\overset{˜}{W}}_{c}^{⊤}\nabla \sigma \left(x\right)G\left(x\right){\left(\nabla \sigma \left(x\right)\right)}^{⊤}{\overset{˜}{W}}_{c}-e_{cH}. \end{array}$$

To update the critic weight vector W^c, the gradient descent algorithm is applied to minimize the objective function:

(23) $$E_{c}=\frac{1}{2}e_{c}^{⊤}e_{c}.$$

The gradient of $E_{c}$ with respect to W^c is given by

(24) ∂Ec∂W^c=ec∇σ(x)f(x)−12∇σ(x)G(x)((∇σ(x))⊤W^c).

Based on (9) and (17), the optimal control policy is obtained by

(25) $$u^{*}\left(x\right)=-\frac{1}{2}R^{-1}g^{⊤}\left(x\right)\left({\left(\nabla \sigma \left(x\right)\right)}^{⊤}W_{c}+\nabla \varepsilon_{c}\left(x\right)\right).$$

Using the estimated critic weights W^c, the approximated optimal control is given by

(26) u^(x)=−12R−1g⊤(x)(∇σ(x))⊤W^c.

Applying the approximate control policy u^(x) to the nominal system (2) yields the closed-loop dynamics

(27) x˙=f(x)−12g(x)R−1g⊤(x)(∇σ(x))⊤W^c.

### 3.3. Stability-Aware Weight Update Mechanism

The conventional critic weight update rule is typically derived under the assumption of an admissible initial control policy. Without a stabilizing initial policy, the learning process may fail to ensure system stability. To address this challenge, we introduce an auxiliary term related to system stability into the update law of the critic network. The enhanced critic weight update law is given by

(28) W^˙c=−αc∂Ec∂W^c+12αsΠ(x,u^(x))∇σ(x)G(x)∇Js(x).

where $\alpha_{c}$ and $\alpha_{s}$ are learning rates. The binary function Π(x,u^(x))∈{0,1} is defined as

(29) Π(x,u^(x))=0,if∇Js(x)⊤(f(x)+g(x)u^(x))<01,otherwise.

**Assumption** **3.** *A continuously differentiable Lyapunov function $J_{s}\left(x\right)$ is selected such that*

(30) $$\;{\dot{J}}_{s}\left(x\right)={\left(\nabla J_{s}\left(x\right)\right)}^{⊤}\dot{x}={\left(\nabla J_{s}\left(x\right)\right)}^{⊤}\left(f\left(x\right)+g\left(x\right)u^{*}\right)<0.$$

*Moreover, there exists a positive definite matrix Λ(x) such that*

(31) $$\;{\left(\nabla J_{s}\left(x\right)\right)}^{⊤}\left(f\left(x\right)+g\left(x\right)u^{*}\right)=-{\left(\nabla J_{s}\left(x\right)\right)}^{⊤}\Lambda \left(x\right)\nabla J_{s}\left(x\right).$$

*Furthermore, there exists a positive constant $\lambda_{s}>0$ satisfying*

(32) $$\;0<\lambda_{s}∥\nabla J_{s}\left(x\right)∥\leq -{\left(\nabla J_{s}\left(x\right)\right)}^{⊤}\dot{x}.$$

**Remark** **2.** Assumption 3 [31] is commonly used for establishing the stability of the closed-loop system under the optimal control $u^{*}$. It relies on the premise that the closed-loop dynamics, $f\left(x\right)+g\left(x\right)u^{*}$, are suitably bounded. Specifically, for some positive constant η>0, it is often assumed that

(33) $$\;∥f\left(x\right)+g\left(x\right)u^{*}∥\leq \eta ∥\nabla J_{s}\left(x\right)∥.$$

*From this, using the Cauchy–Schwarz inequality, we have*

$$∥{\left(\nabla J_{s}\left(x\right)\right)}^{⊤}\left(f\left(x\right)+g\left(x\right)u^{*}\right)∥\leq ∥\nabla J_{s}\left(x\right)∥∥f\left(x\right)+g\left(x\right)u^{*}∥\leq \eta ∥\nabla J_{s}{\left(x\right)∥}^{2}.$$

*The specific structure (31) provides a concrete way to ensure ${\dot{J}}_{s}\left(x\right)<0$ (for $\nabla J_{s}\left(x\right)\neq 0$) and is consistent with this derived upper bound. Such an assumption is generally considered reasonable, and $J_{s}\left(x\right)$ is often chosen as a quadratic function in practice.*

The auxiliary stabilizing term provides real-time feedback on the stabilizing capability of the current policy based on the Lyapunov stability theorem and adjusts the gradient direction accordingly. It is worth noting that the binary function Π(x,u^(x)) is defined based on Lyapunov stability conditions. When the nonlinear system is stable—i.e., when the time derivative of the Lyapunov function satisfies ∇Js(x)⊤f(x)+g(x)u^<0—the auxiliary term is inactive, and the critic update is driven purely by minimizing the approximation error. In contrast, when the system tends to diverge, the auxiliary term is activated to redirect the learning process toward a stabilizing control policy. This modification provides a structural enhancement to conventional critic learning, enabling the critic to converge eventually without requiring any admissible initial controller. It significantly reduces sensitivity to initial conditions and improves the algorithm’s practicality in real-time control scenarios. The theoretical validity of this mechanism will be formally established in the next section using Lyapunov-based analysis.

**Remark** **3.** *In ADP, PI typically relies on an IAP to maintain closed-loop stability, whereas VI is more flexible but often lacks a verifiable stability guarantee during purely online learning. To address this issue, we introduce in (28) a Lyapunov-criterion-based stability switch Π(x,u^(x)). When the update direction may cause ${\dot{J}}_{s}\left(x\right)\geq 0$, the switch automatically activates an auxiliary update, which integrates HJB optimization and the stability requirement during learning within a single, unified framework.*

Within the assumptions adopted in this paper and over the working domain Ω, the design enables online optimization without an IAP and provides a proven safety guarantee; Section 3.4 establishes UUB. Compared with multi-stage or hybrid schemes [7,8], the proposed method embeds learning-phase stability directly into the update law, thereby delivering a verifiably safeguarded online learning process. This capability remains relatively uncommon among existing IAP-free ADP frameworks and is well aligned with the real-time control demands of safety-critical systems.

### 3.4. Fault Compensation Design

Based on the analysis in Section 3.1, we further propose a fault compensation control structure to mitigate the influence of the actuator fault. To this end, we propose the following fault compensation control law:

(34) ua(x)=u^(x)+f^a.

**Remark** **4.** *The proposed control scheme belongs to the category of active FTC. By incorporating a dynamically estimated fault compensation term, the controller can adapt to real-time fault variations and maintain fault-tolerant performance.*

**Figure 1:**
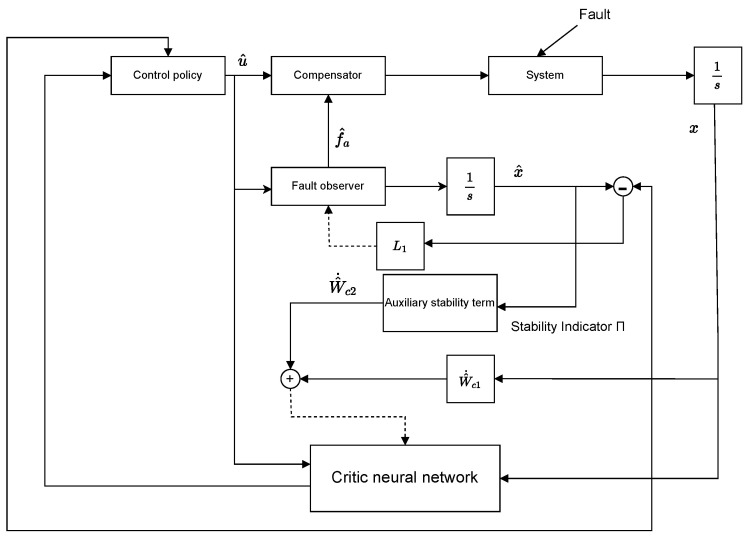
The structural diagram of the observer-based ADP scheme with auxiliary stabilizing term for FTC.

The structural diagram of the observer-based ADP scheme with the auxiliary stabilizing term for FTC is shown in Figure 1.

### 3.5. Stability Analysis

**Assumption** **4.** *There exist positive constants $\lambda_{\phi },\lambda_{A},\lambda_{1},\lambda_{e},\lambda_{G},\lambda_{4},$ and $\lambda_{6}$ such that for all x∈Ω, $∥\phi \left(x\right)∥\leq \lambda_{\phi }$, $∥A\left(x\right)∥\leq \lambda_{A}$, $∥AW_{c}∥\leq \lambda_{1}$, $∥e_{cH}∥\leq \lambda_{e}$, $∥G\left(x\right)∥\leq \lambda_{G}$, $∥\nabla \sigma \left(x\right)∥\leq \lambda_{4}$, and $∥\nabla \varepsilon_{c}\left(x\right)∥\leq \lambda_{6}$ [31].*

**Theorem** **2.** *Consider the system described by Equation (2). If the feedback control law is implemented as (26), and the critic network weights are updated according to the learning rule (28), then the closed-loop system state x and the weight estimation error ${\overset{˜}{W}}_{c}$ are UUB.*

**Proof.**  To establish the stability analysis, we define a Lyapunov candidate function as

(35) $$L=\frac{1}{2\alpha_{c}}{\overset{˜}{W}}_{c}^{⊤}{\overset{˜}{W}}_{c}+\frac{\alpha_{s}}{2\alpha_{c}}J_{s}\left(x\right).$$

Taking the time derivative of *L*, we obtain

(36) $$\dot{L}=\frac{1}{\alpha_{c}}{\overset{˜}{W}}_{c}^{⊤}{\dot{\overset{˜}{W}}}_{c}+\frac{\alpha_{s}}{2\alpha_{c}}{\left(\nabla J_{s}\left(x\right)\right)}^{⊤}\dot{x}.$$

Substituting the critic weight update law, the dynamics of the weight estimation error are given by

(37) W˜˙c=αc∂Ec∂W^c−12αsΠ(x,u^(x))∇σ(x)g(x)R−1g⊤(x)∇Js(x).

Substituting (37) and (27) into (36) yields

(38) L˙=−W˜c⊤ϕ(x)+14W˜c⊤A(x)W˜c−12W˜c⊤A(x)Wc+ecH×W˜c⊤ϕ(x)+12W˜c⊤A(x)W˜c−12W˜c⊤A(x)Wc−αs2αcΠ(x,u^(x))W˜c⊤∇σ(x)g(x)R−1g⊤(x)∇Js(x)+αs2αc(∇Js(x))⊤x˙,

where

$$\begin{array}{cc} A(x) & =\nabla \sigma \left(x\right)g\left(x\right)R^{-1}g^{⊤}\left(x\right){\left(\nabla \sigma \left(x\right)\right)}^{⊤},\\ \phi (x) & =\nabla \sigma (x)f(x). \end{array}$$

Based on Assumption 4, we have

(39) L˙≤−18−38ϕ12−316ϕ22λA2W˜c4+12λAλe+1+38ϕ12λϕ2+34+316ϕ22λ12W˜c2+34λe2−αs2αcΠx,u^W˜c⊤∇σxgxR−1g⊤x∇Jsx+αs2αc∇Jsx⊤x˙.

□

Case 1: When Π(x,u^)=0, ${\left(\nabla J_{s}\left(x\right)\right)}^{⊤}\dot{x}<0$. According to Assumption 3, there exists a positive constant $\lambda_{s}$ such that the inequality $0<\lambda_{s}∥\nabla J_{s}\left(x\right)∥\leq -{\left(\nabla J_{s}\left(x\right)\right)}^{⊤}\dot{x}$ holds. Thus, (39) can then be expressed as

(40) $$\;\begin{array}{cc} \dot{L}\leq \;\; & -\lambda_{2}{∥{\overset{˜}{W}}_{c}∥}^{4}+\lambda_{3}{∥{\overset{˜}{W}}_{c}∥}^{2}\\ \; & +\frac{3}{4}\lambda_{e}^{2}-\frac{\alpha_{s}}{{2\alpha }_{c}}\lambda_{s}\left\|\nabla J_{s}\left(x\right)\right\|, \end{array}$$

where

$$\begin{array}{cc} \;\lambda_{2} & =\left(\frac{1}{8}-\frac{3}{8}\phi_{1}^{2}-\frac{3}{16}\phi_{2}^{2}\right)\lambda_{A}^{2},\\ \lambda_{3} & =\frac{1}{2}\lambda_{A}\lambda_{e}+\left(1+\frac{3}{8\phi_{1}^{2}}\right)\lambda_{\phi }^{2}+\left(\frac{3}{4}+\frac{3}{16\phi_{2}^{2}}\right)\lambda_{1}^{2}. \end{array}$$

Therefore, it follows that $\dot{L}<0$ when either of the following conditions is satisfied:

(41) $$∥{\overset{˜}{W}}_{c}∥\geq \sqrt{\frac{\lambda_{3}+\sqrt{3\lambda_{e}^{2}\lambda_{2}+\lambda_{3}^{2}}}{2\lambda_{2}}}≜A_{1}$$

or

(42) $$∥\nabla J_{s}\left(x\right)∥\geq \frac{1}{\lambda_{s}}\left(\frac{2\alpha_{c}}{\alpha_{s}}\right)\left(\frac{\lambda_{3}^{2}}{4\lambda_{2}}+\frac{3}{4}\lambda_{e}^{2}\right)≜B_{1}.$$

Case 2: When Π(x,u^(x))=1, the auxiliary term is active. According to (26) and (25), the difference between the optimal control and its estimate is given by

(43) u*(x)−u^(x)=−12R−1g⊤(x)∇σ(x)⊤W˜c+∇εc(x).

Following the previous derivation, we have x˙=f(x)+g(x)u^(x)=f(x)+g(x)u*(x)+g(x)(u^(x)−u*(x)). Substituting the control error term (43) into the expression of $\dot{x}$ yields

(44) $$\dot{x}=f\left(x\right)+g\left(x\right)\left(u^{*}+\frac{1}{2}R^{-1}g^{⊤}\left(x\right)\left({\left(\nabla \sigma (x)\right)}^{⊤}{\overset{˜}{W}}_{c}+\nabla \varepsilon_{c}\left(x\right)\right)\right).$$

Therefore, the Lyapunov function derivative now becomes

(45) $$\begin{array}{c} \begin{array}{cc} \dot{L}⩽ & -\left(\frac{1}{8}-\frac{3}{8}\phi_{1}^{2}-\frac{3}{16}\phi_{2}^{2}\right)\lambda_{A}^{2}{∥{\overset{˜}{W}}_{c}∥}^{4}\\ & +\left(\frac{1}{2}\lambda_{A}\lambda_{e}+\left(1+\frac{3}{8\phi_{1}^{2}}\right)\lambda_{\phi }^{2}+\left(\frac{3}{4}+\frac{3}{16\phi_{2}^{2}}\right)\lambda_{1}^{2}\right){∥{\overset{˜}{W}}_{c}∥}^{2}\\ & +\frac{3}{4}\lambda_{e}^{2}+\frac{\alpha_{s}}{2\alpha_{c}}{\left(\nabla J_{s}\left(x\right)\right)}^{⊤}\left(f\left(x\right)+g\left(x\right)u^{*}\right)\\ & -\frac{\alpha_{s}}{4\alpha_{c}}{\overset{˜}{W}}_{c}^{⊤}\nabla \sigma \left(x\right)g\left(x\right)R^{-1}g^{⊤}\left(x\right)\nabla J_{s}\left(x\right)\\ & +\frac{\alpha_{s}}{4\alpha_{c}}{\left(\nabla J_{s}\left(x\right)\right)}^{⊤}g\left(x\right)R^{-1}g^{⊤}\left(x\right)\nabla \varepsilon_{c}\left(x\right). \end{array} \end{array}$$

Substituting (31) from Assumption 3 into (45), and applying the Cauchy–Schwarz and Young’s inequalities to the penultimate term of the above equation yields

$$\begin{array}{cc} -\frac{\alpha_{s}}{4\alpha_{c}}{\overset{˜}{W}}_{c}^{⊤}\nabla \sigma \left(x\right)g\left(x\right)R^{-1}g^{⊤}\left(x\right)\nabla J_{s}\left(x\right) & \leq \frac{\alpha_{s}}{8\alpha_{c}}∥{\overset{˜}{W}}_{c}{∥}^{2}+\frac{\alpha_{s}\lambda_{4}^{2}\lambda_{G}^{2}}{8\alpha_{c}}{∥\nabla J_{s}\left(x\right)∥}^{2}, \end{array}$$

Thus, we have

(46) $$\begin{array}{cc} \dot{L}\leq & -\lambda_{2}∥{\overset{˜}{W}}_{c}{∥}^{4}+\left(\lambda_{3}+\frac{\alpha_{s}}{8\alpha_{c}}\right){∥{\overset{˜}{W}}_{c}∥}^{2}\\ \; & +\frac{3}{4}\lambda_{e}^{2}-\lambda_{5}{∥\nabla J_{s}\left(x\right)∥}^{2}\\ \; & +\frac{\alpha_{s}}{4\alpha_{c}}\lambda_{G}\lambda_{6}∥\nabla J_{s}\left(x\right)∥, \end{array}$$

where $\lambda_{5}=\frac{\alpha_{s}}{2\alpha_{c}}\left(\lambda_{\min}\left(\Lambda \right)-\frac{1}{4}\lambda_{G}^{2}\lambda_{4}^{2}\right)$.

Therefore, it follows that $\dot{L}<0$ if either

(47) $$\;∥{\overset{˜}{W}}_{c}∥⩾\sqrt{\frac{8\alpha_{c}\lambda_{3}+\alpha_{s}}{16\alpha_{c}\lambda_{2}}+\sqrt{\frac{{\left(8\alpha_{c}\lambda_{3}+\alpha_{s}\right)}^{2}}{256\alpha_{c}^{2}\lambda_{2}^{2}}+\frac{3\lambda_{e}^{2}}{4\lambda_{2}}+\frac{\alpha_{s}^{2}\lambda_{G}^{2}\lambda_{6}^{2}}{64\alpha_{c}^{2}\lambda_{2}\lambda_{5}}}}≜A_{2}$$

or

(48) $$∥\begin{array}{c} \nabla J_{s}\left(x\right) \end{array}∥\geq \frac{\alpha_{s}\lambda_{G}\lambda_{6}}{8\alpha_{c}\lambda_{5}}+\sqrt{\frac{{\left(8\alpha_{c}\lambda_{3}+\alpha_{s}\right)}^{2}}{256\alpha_{c}^{2}\lambda_{2}\lambda_{5}}+\frac{3\lambda_{e}^{2}}{4\lambda_{5}}+\frac{\alpha_{s}^{2}\lambda_{G}^{2}\lambda_{6}^{2}}{64\alpha_{c}^{2}\lambda_{5}^{2}}}≜B_{2}.$$

By combining the two cases above, we have $\dot{L}<0$ if the following condition holds:

(49) $$\;∥{\overset{˜}{W}}_{c}∥>\max\left(A_{1},A_{2}\right)\;\mathrm{or}\;∥\nabla J_{s}\left(x\right)∥>\max\left(B_{1},B_{2}\right).$$

According to the standard Lyapunov stability theorem, it follows that both the system state *x* and the critic weight estimation error ${\overset{˜}{W}}_{c}$ are UUB. This concludes the proof.

## 4. Simulation

This section demonstrates the effectiveness of the proposed stability-guaranteed ADP-based FTC through two simulation examples.

### 4.1. Example 1

Consider the following nonlinear affine system:

(50) $$\;\dot{x}=\left[\begin{array}{c} x_{2}-x_{1}\\ \begin{array}{c} -0.5x_{1}-0.5x_{2}+\\ 0.5x_{2}{\left(\cos\left(2x_{1}\right)+2\right)}^{2} \end{array} \end{array}\right]+\left[\begin{array}{c} 0\\ \cos(2x_{1})+2 \end{array}\right]\left(u\left(t\right)-f_{a}\left(t\right)\right),$$

with the initial state $x\left(0\right)={\left[0.5,-0.5\right]}^{T}$. $f_{a}\left(t\right)$ represents the unknown actuator fault, which is defined as

(51) $$f_{a}\left(t\right)=\left\{\begin{array}{cc} 0.2\cos\left(\frac{t}{2\pi }\right), & 30\;s\leq t\leq 60\;s\\ 0, & \mathrm{otherwise}. \end{array}\right.$$

The parameters of the performance index function are selected as $Q=2I_{2}$ and R=1, respectively, where $I_{2}$ is the 2×2 identity matrix. The initial state of the observer is set to x^(0)=[0.5,−0.5]T, and the initial value of the fault estimation is set to f^a(0)=0. The observer gain is chosen as $L_{1}=43I_{2}$ and the learning rate of the fault observer is set to $L_{2}=100$. A critic NN is constructed to approximate the value function. The weight vector is W^c=[W^c1,W^c2,W^c3]T with initial weights W^c(0)=[0.7,0.9,0.6]T, and the activation function of the critic NN is $\sigma \left(x\right)=\left[x_{1}^{2},x_{1}x_{2},x_{2}^{2}\right]$. The learning rate for the critic network is set to $\alpha_{c}=1$. The parameter of the auxiliary stabilizing term is set as $\alpha_{s}=1$. The total simulation duration was 100 s, and the actuator fault $f_{a}\left(t\right)$ affects the system during the interval t∈[30s,60s].

**Figure 2:**
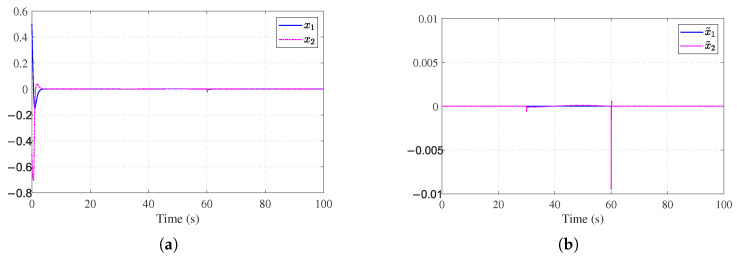
State estimation performance for Section 4.1. (**a**) State performance for Section 4.1. (**b**) Observer estimation error for Section 4.1.

**Figure 3:**
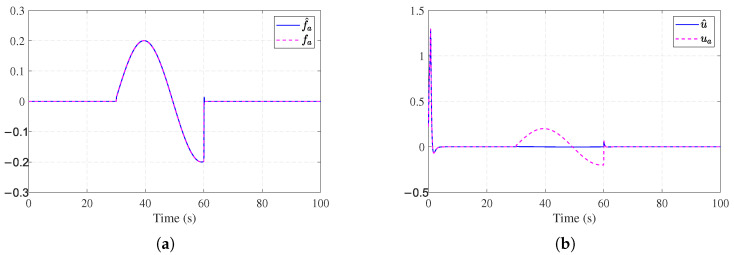
Fault estimation and control performance for Section 4.1. (**a**) Actuator fault estimation for Section 4.1. (**b**) Control input signals for Section 4.1.

**Figure 4:**
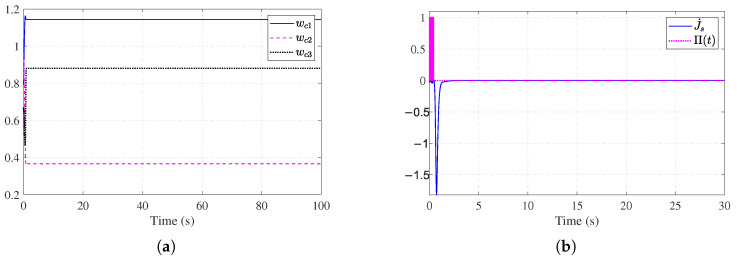
Learning dynamics and stability mechanism for Section 4.1. (**a**) Critic NN weights for Section 4.1. (**b**) Stability mechanism performance for Section 4.1.

**Figure 5:**
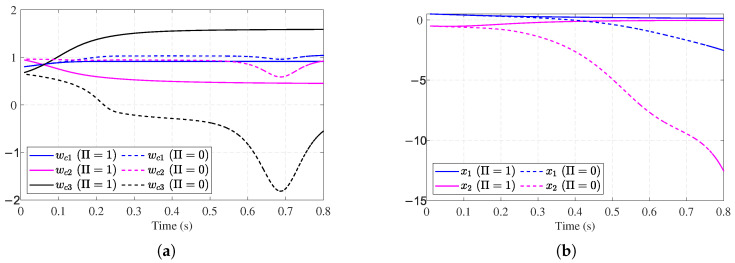
Performance without stability mechanism for Section 4.1. (**a**) Critic NN without stability mechanism for Section 4.1. (**b**) System state trajectories without stability mechanism for Section 4.1.

Simulation results are shown in Figure 2, Figure 3, Figure 4 and Figure 5. The state trajectory performance is shown in Figure 2a. It shows that the states converge within 5 s. Figure 2b shows the time response curves of the observer estimation errors ${\overset{˜}{x}}_{1}$ and ${\overset{˜}{x}}_{2}$. As illustrated in Figure 2b, the state observation errors converge to a small neighborhood around the origin. Figure 3a presents the curves of the actual actuator fault $f_{a}\left(t\right)$ and the fault estimation f^a(t). The results indicate that the designed fault observer can accurately identify the unknown fault signal rapidly after fault occurrence. Figure 3b shows the control signals of the nominal optimal control (26) and the ADP-based FTC policy (34). It can be observed that after the fault occurs, the ADP-based FTC policy adjusts accordingly. Figure 4a shows the evolution process of the critic NN weights W^c1(t), W^c2(t), and W^c3(t), which converge to [1.14, 0.36, 0.88]. Figure 4b further shows the working mechanism of the auxiliary stabilizing term, including the stability condition ${\dot{J}}_{s}\left(t\right)$ and the stability indicator Π(t). It can be seen that the stability indicator Π(t) is frequently activated in the early learning phase and eventually converges to 0.

To further verify the effectiveness of the proposed auxiliary stabilizing term, a comparative analysis is performed against the conventional ADP method presented in [30]. The update law in [30] relies on a standard gradient descent approach, which typically requires an initial admissible policy to ensure stability. By removing our auxiliary stabilizing term, we effectively simulate this conventional approach under conditions where the initial policy may not be stabilizing. Figure 5a,b show the divergent critic NN weights and system states without the auxiliary stabilizing term, which indicates that the initial control fails to stabilize the system. Fortunately, with the auxiliary stabilizing term, the training process of the weight vector is reinforced until the system exhibits stable behavior (see Figure 4a,b). Thus, the requirement of an initial stabilizing control is relaxed.

### 4.2. Example 2

In this case, we consider a nonlinear mass-spring-damper system, with dynamics described as follows:

(52) $$\dot{x}=\left[\begin{array}{c} x_{2}\\ -0.02x_{1}-0.67x_{1}^{3}-0.1x_{2}^{3} \end{array}\right]+\left[\begin{array}{c} 0\\ 1 \end{array}\right]\left(u\left(t\right)-f_{a}\left(t\right)\right),$$

with the initial state $x\left(0\right)={\left[0.5,-0.5\right]}^{T}$. The control input u(t) is defined as in Example 1. In this case, the actuator fault $f_{a}\left(t\right)$ is chosen as a different model, specifically as follows:

(53) $$f_{a}\left(t\right)=\left\{\begin{array}{cc} 0.1\sin\left(\frac{1}{\pi }t\right), & 40\;s\leq t\leq 70\;s\\ 0, & \mathrm{otherwise}. \end{array}\right.$$

The observer gain is chosen as $L_{1}=5I_{2}$ and the learning rate of the fault observer is set to $L_{2}=100$. The weight vector is W^c=[W^c1,W^c2,W^c3]T with initial weights selected as W^c(0)=[0.03,0.2,0.04]T. The learning rate is $\alpha_{c}=3$. The total simulation duration was still 100 s, with the actuator fault $f_{a}\left(t\right)$ affecting the system during the interval t∈[40s,70s]. Other parameters and initial conditions are chosen the same as Example 1.

**Figure 6:**
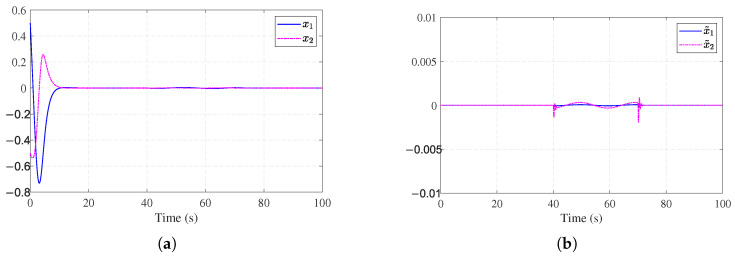
State estimation performance for Section 4.2. (**a**) State performance for Section 4.2. (**b**) Observer estimation errors for Section 4.2.

**Figure 7:**
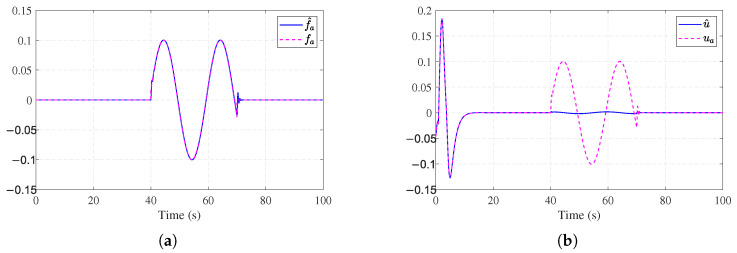
Fault estimation and control performance for Section 4.2. (**a**) Actuator fault estimation for Section 4.2. (**b**) Control input signals for Section 4.2.

**Figure 8:**
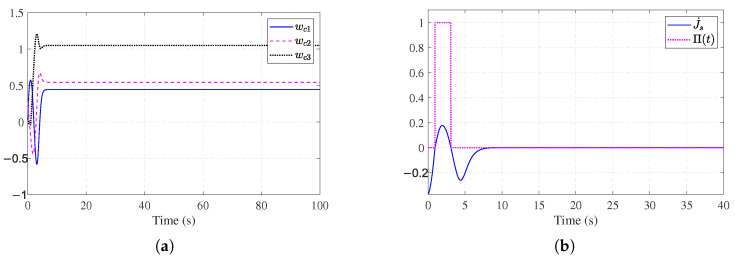
Learning dynamics and stability mechanism for Section 4.2. (**a**) Critic NN weights for Section 4.2. (**b**) Stability mechanism performance for Section 4.2.

**Figure 9:**
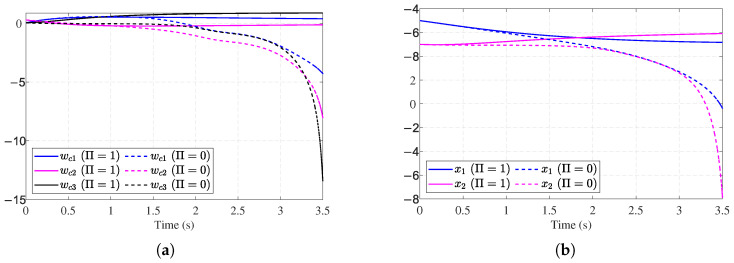
Performance without stability mechanism for Section 4.2. (**a**) Critic NN weights without stability mechanism for Section 4.2. (**b**) System state trajectories without stability mechanism for Section 4.2.

Simulation results for Example 2 are shown in Figure 6, Figure 7, Figure 8 and Figure 9. Figure 6a shows the trajectories of the stable system states. Figure 6b shows the convergent observer errors. Figure 7a presents the curves of the actual actuator fault and its estimation. It can be observed that the fault observer can estimate the actuator fault, verifying the effectiveness of the fault observer. Figure 7b demonstrates the nominal control and ADP-based FTC signals. Figure 8a shows the trajectories of the critic weights converging to [0.44, 0.54, 1.04]. Figure 8b illustrates the working condition of the auxiliary stabilizing term. The stability indicator is mainly triggered in the early stages and eventually converges to 0. Figure 9a,b show that without the auxiliary stabilizing term, the critic NN weights and system trajectories diverge, indicating that the control policy fail to stabilize the system. Figure 6a and Figure 8a together with Figure 9a,b verify that the stability-aware Weight update mechanism can effectively overcome the dependency on an initial admissible control under different models.

## 5. Conclusions

This paper presents a stability-guaranteed ADP-based FTC scheme for nonlinear systems with the actuator fault, which eliminates the dependency on an initial admissible policy. This is primarily achieved by embedding a Lyapunov-based stability condition directly into the critic network’s learning process. In parallel, the actuator fault is estimated by an observer, and compensated based on the nominal optimal control, which enhances the system’s resilience. We have rigorously proven the UUB stability of all signals, including the closed-loop system states, the estimation error of critic NN weights and the observer error, and demonstrated the scheme’s effectiveness through simulations. Future research will focus on extending this framework to more complex scenarios. Key directions include designing observers for intermittent faults, developing data-driven strategies for systems with unknown dynamics, and applying the method to distributed networked architectures to address challenges such as asynchronous group consensus [32], containment control with deferred constraints [33], and resilience against cyber-attacks via event-triggered control [34].

## Data Availability

The data presented in this study are available within the article.
